# Community‐Based System Dynamics for Mobilizing Communities to Advance School Health

**DOI:** 10.1111/josh.12961

**Published:** 2020-11-12

**Authors:** Ellis Ballard, Allison Farrell, Michael Long

**Affiliations:** ^1^ Assistant Professor of Practice, Director, (eballard@wustl.edu), Social System Design Lab, Brown School, Washington University, 1 Brookings Drive, Campus Box 1196, St. Louis, MO 63130. USA; ^2^ K‐12 Education Program Coordinator, (allison.farrell@wustl.edu), Social System Design Lab, Brown School, Washington University in St. Louis, 1 Brookings Drive, Campus Box 1196, St. Louis, MO 63130. USA; ^3^ Assistant Professor, (michael_long@gwu.edu), Department of Prevention and Community Health, George Washington University Milken School of Public Health, 950 New Hampshire Avenue, NW, Washington, DC 20052. USA

**Keywords:** school health, systems thinking, behavioral health, stakeholder engagement, system dynamics, WSCC model

## Abstract

**BACKGROUND:**

Frameworks such as the WSCC model provide evidence‐based guidance for addressing school health at the school, district, and regional level. However, frameworks do not implement themselves; they require the mobilization and collaboration of stakeholders within communities and an understanding of the unique resources and barriers within each context. Furthermore, addressing school health presents a complex systems problem.

**METHODS:**

Community‐based system dynamics (CBSD) is a participatory approach for engaging communities in understanding and changing complex systems. We used a descriptive multiple case study design to evaluate how and why CBSD was used as a tool for stakeholders to engage with the complexity of school health.

**RESULTS:**

We analyzed 3 cases to understand how these methods were used to enhance collaboration, analysis, and community action at multiple levels, including in 2 school districts, with a city‐wide stakeholder committee, and with a group of high school students.

**CONCLUSIONS:**

Community‐based system dynamics presents a promising approach for building shared language and ownership among stakeholders, tailoring to local community contexts, and mobilizing stakeholders for action based on new system insights. We close with a discussion of unique opportunities and challenges of expanding the use of CBSD in the field of school health.

The Whole School, Whole Community, Whole Child (WSCC) framework emphasizes the need for stakeholders to have a common understanding of the interrelatedness of learning and health prior to policy or programmatic changes; success is also predicated on schools and communities having a shared responsibility for the health and education of children.^1^ Whereas the WSCC framework illustrates the interconnection of components necessary for centering school health on the child, a framework is not an intervention.

In practice, public health interventions in schools are hindered by limited impact and sustainability, with challenges of commitment of senior leaders, staff buy‐in, human resource turnover, and changes in prioritization of health and education outcomes.^2^ Even when schools are committed to adequately resourcing all of the components of the WSCC framework, this does not guarantee work across silos. In states that have “broad and deep coverage” of the 10 WSCC domains, there is little integration between domains and topic areas.^3^ Discussion of the WSCC framework acknowledges that implementation may vary across districts and schools based upon the starting conditions within communities including school leadership, policies, culture, school and community needs and assets, staff availability, time, financial resources, family engagement, and community involvement.^4^ Understanding this contextual specificity requires student voice, ownership, and leadership,^5^ and establishing interest and buy‐in from diverse stakeholders in order to generate authentic and meaningful insight related to the needs and assets of the school and overall community.^4^

The challenge of understanding and acting to promote school health has been conceptualized as a complex systems problem with features such as interventions that are community context dependent, delays and gaps in information flows, formal and informal feedback mechanisms balancing and resisting change, and a pattern of learning, change, and evolution that mean that what may work at one point in time may be obsolete at another.^6^, ^7^, ^8^, ^9^ This observation echoes arguments in population health communities that argue that messy public health problems challenges are resistant to change precisely because of their complexity, and call for an embrace in systems modeling tools to formally represent and design policy interventions.^10^ Proponents of a complex systems view of schools argue that this perspective may help explain some of the challenges of introducing and sustaining change in schools, and can lead us to adopt more sophisticated approaches to diffusing programs that accounts for the diverse, complex, and context‐specific nature of individual school systems.^9^

The system dynamics perspective explores complex system behavior through a feedback lens, conceiving a school system or a community as an interconnected set of parts, components, stakeholders and functions that are structured in such a way to create behavior—in this case student health—over time. Whereas the system may be buffeted by outside factors—policy changes, interventions, economic shocks, community change, the system's response to these factors is a product of the underlying system structure.^11^ System dynamics provides a formal method for building qualitative causal maps and formal models with computer simulation to describe the underlying system structure creating the system behavior we see,^12^ and to more formally discuss ways to change that structure to realize our desired future. Figure 1 presents a simple 2‐loop casual loop diagram that illustrates this feedback perspective around the issue of initiative fatigue. This simple, stylized diagram describes common traps in district approaches to addressing school culture challenges through the common system structure, or archetype, called “fixes that fail.”^13^, ^14^ School districts implement new interventions or policy initiatives but, because of the delay realizing the impact of these interventions, district leadership may continue to cycle through new interventions in an effort to find an intervention that sticks. An underappreciated feedback loop is that this accumulation of attempted (and aborted) interventions diminishes staff buy‐in for implementation of future interventions, diminishing implementation effectiveness, and thus, weakening the overall impact of subsequent interventions.

**Figure 1:**
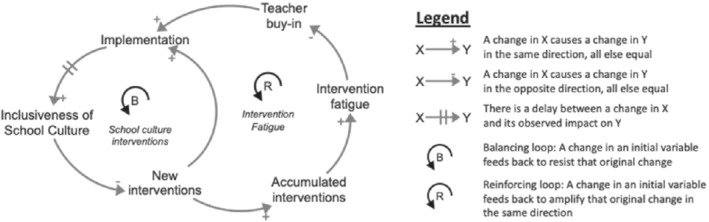
Illustration of the Feedback Perspective

Community‐based system dynamics (CBSD) is one approach that employs participatory methods for system dynamics modeling.^15^ CBSD engages stakeholders who are most implicated in policy decisions to build their own model representations of system structure generating a problem, often using structured group model building processes of scripted modeling exercises to facilitate the co‐development of causal maps and qualitative models.^16^ What distinguishes CBSD tradition from other participatory system dynamics methods is an explicit emphasis on the development of system dynamics and systems thinking capabilities among participants around the use of system dynamics models, their assumptions, and limitations. The goal of CBSD is to increase ownership of the system insights within communities by the people most directly impacted by policy decisions and to sustain their use after formal modeling projects or collaborations have ended.^17^

Although participatory approaches are not new to school health,^18^, ^19^ the complexity involved in introducing and sustaining change in school systems suggests the value of a participatory process that engages with this complexity in order to generate ownership and buy‐in, while at the avoiding the common traps of policy interventions: pushing on levers that fail to create change; developing interventions or policy proposals that address symptoms, not causes; or, worse, designing well‐intentioned interventions that make system inequities worse rather than better.^20^

In this paper, we describe CBSD as a specific approach to system dynamics modeling that leverages community voice and insight about complex system structure to advance collaboration, analysis, and action. We use a descriptive multiple case study design to evaluate how using CBSD methods supports community efforts to address school health as a systems issue in order to achieve the goals of the WSCC framework.

## METHODS

### Study Design and Research Questions

We used a descriptive multiple case study design with the goal of developing new research questions about the use of CBSD to promote school health.^21^, ^22^ Data collection and analysis for each case began separately, with a limited set of research questions and objectives co‐created with participants in the CBSD process. Each case had an intrinsic focus, meaning the specific issues within the context of each case were of primary interest for data collection and analysis.^23^ In line with Stake's use of the concept of “progressive focusing,” the study design and research questions for this multiple case study evolved into an instrumental focus on the issue of how CBSD can be used to support school health. Whereas the evolution in focus is align with exploratory designs, the primary purpose of this descriptive study was not theory generation. The current descriptive multiple case analysis addresses the following research questions:

1. How did participants integrate systems thinking into their understanding of school health problems and solutions?
2. How did participants develop common language during CBSD workshops?
3. How did the CBSD methods support integration of broader understandings of the WSCC framework with the complexity of the local school context?
4. How did the use of CBSD methods support mobilization for action?

### Case Selection

To illuminate the potential for contribution of CBSD methods to the work of promoting school health for the whole child, we selected 3 cases from a body of work applying CBSD approaches to topics of school health in K‐12 education contexts. We used a purposive diverse case selection method to include cases that maximize variability across dimensions of applied and theoretical interest.^24^ Cases were selected to include variation within 2 dimensions: (1) the definition of community (ie, multiple stakeholders within an urban school district, a stakeholder learning community within a city, and a community of youth from across a region) and (2) dimensions of school health (ie, overall wellbeing, mental health, and emotional wellbeing). The theoretical importance of selecting cases for diversity for this descriptive study was balanced against pragmatic limitations based on the range of projects conducted by the team, estimated at 20–25 school‐based CBSD and group model building projects, including supervised student work. Although we have considered the representativeness of these cases post hoc and believe that these cases are representative of the experience of school communities in many cities in the United States, the projects all began with an intrinsic focus on each case instead of on generalizability.

### Data Collection and Analysis

The primary mode of data collection within these CBSD case studies is participant observation. CBSD projects are led by a community‐driven Core Modeling Team that is responsible for the design decisions, informing selection and recruitment of participants, and tailoring problem framing and session prompts.^17^ Core modeling teams are composed of 3–5 participants, representing several distinct perspectives:

- Community voice perspectives to consider language and power dynamics.
- System dynamics modeling perspective to inform the design and coherence of modeling work.
- Group dynamics perspectives to account for interpersonal conflict and group facilitation process.
- Organizational or substantive perspectives to tailor the design for relevance to the specific programmatic or policy contexts.

Following conventions of group model building practice in system dynamics, community workshops are structured through multiple sessions in which initial model structure is generated by participants through scripted modeling activities or “scripts.”^25^, ^26^, ^27^ That structure is refined or synthesized by members of the core modeling team, then it is presented back to community participants in a subsequent workshop for real‐time critique and refinement, or as an input to subsequent modeling activities. The specific sequencing of group model building scripts vary depending on the design determined by the core modeling team, though workshops frequently follow principles of alternating convergent and divergent scripts in which the outputs from one session serves as an input for the next.^28^ This iterative process provides a structure for integrating multiple stakeholder group model outputs into a single synthesis model, then bringing that synthesis back to stakeholder groups either together or separately to review, critique, and revise. This process means that the productive work of model review occurs in primarily community contexts rather than having the synthesis and interpretation take place within the project team alone.

The structured CBSD process includes extensive documentation of participant‐generated artifacts and detailed minutes from ongoing meetings with participants. We compiled artifacts and other forms of evidence for each of the 3 cases using the structured CBSD protocols. The database of artifacts includes notes from core modeling planning meetings, workshop agendas and facilitation manuals, photos of outputs from workshop activities, process photos, transcribed workshop notes, and synthesized models with group revisions. Workshops in all 3 case studies adapted and utilized some combination of the following “scripts” from Scriptapedia, an online repository of structured group model building activities:^27^ Hopes and Fears, Behavior Over Time Graphs, Connection Circles, Causal Mapping in Small Groups, Model Review, and Action Ideas. Cases may also include quantitative survey data or collection of secondary quantitative data. Table 1 provides an overview of artifacts generated from the 3 workshops.

**Table 1:** Compilation of Project Artifacts

	Case 1: Healthy Schools in St. Louis, MO	Case 2: Behavioral Health Improvement in Washington, DC	Case 3: Youth Summit for Emotional Support in St. Louis, MO
Planning documents	Notes from 8 Core Modeling Team meetings 4 Facilitation manuals	Notes from 4 Core Modeling Team meetings 2 Reference modes 2 Facilitation manuals	Notes from 6 Youth Planning Meetings 1 Facilitation manual 1 Reference mode
Workshop artifacts	35 Hopes and Fears 30 Behavior Over Time Graphs 5 Connection Circles 5 Small Group Causal Loop Diagrams 18 Action Ideas 4 Workshop Notes 2 Synthesized Causal Loop Diagrams with Group Revisions	33 Hopes and Fears 18 Behavior Over Time Graphs 4 Small Group Causal Loop Diagrams 1 Synthesized Causal Loop Diagram with group revisions 2 Workshop Notes	41 Hopes and Fears 22 Behavior Over Time Graphs 6 Connection Circles 5 Small Group Causal Loop Diagrams 6 Small Group Stock and Flow Diagrams 25 Action ideas 4 Calls to Action
Post‐workshop artifacts	1 Synthesized Causal Loop Diagram Process summary slide deck	1 Synthesized Causal Loop Diagram Process summary slide deck	1 Synthesized Stock and Flow Diagram 18 Participant Post Surveys 1 Policy brief Process summary slide deck

In contrast to Yin's positivist approach to case study research, our approach to the design of this study employs a constructivist epistemological stance focused on the process by which knowledge is constructed. Through the structured CBSD process, the resulting causal loop diagrams (CLDs) and models function as a socially constructed hypothesis of the structure underlying the success or failure of school health initiatives. These models function as *boundary objects* that allow for each stakeholder perspective to see their mental model reflected in the map, while maintaining the ability to modify and critique that map.^29^ The use of the CLD as a boundary object in CBSD embeds data collection, analysis, and confidence building in model structure within co‐created research plans with the Core Modeling Team, which supports the rigor and coherence of case study research. Whereas CBSD uses a qualitative approach to develop a shared understanding of systems dynamics within a community context, the qualitative methods used here are distinct from the frequently employed qualitative research methods of data collection and analysis using coding of data from artifacts (eg, interview transcripts, images, memos, etc.) and thematic analysis. The use of structured protocols and collection of evidence databases supports the reliability of the study by creating a documentary record through which analysis can be compared to artifacts and session notes to confirm the correspondence of analysis to statements or themes from the sessions. The multisite nature of this study substantially enhances the external validity of any findings from cross‐case pattern matching.

Each within‐case analysis includes a description of the specific community and problem context, the structure of CBSD activities undertaken under the umbrella of the project, insights into strategic or structural challenges in school health, and provisional descriptions of impact, understanding that all 3 are ongoing as of the time of writing. For the cross‐case analysis, we used a site‐ordered descriptive matrix addressing each of the research questions to identify cross‐cutting patterns (Table 2). For this analysis, we used each of the study's research questions to interrogate the respective CLD as the primary synthesis from each of the 3 cases. We then checked our interpretations against the other artifacts from each case and discussed our findings among the research team. We discussed the cross‐case synthesis as a team to identify similarities and differences in how CBSD functioned to promote school health.

**Table 2:** Descriptive Analysis of Community‐Based Systems Dynamics from 3 School Health Cases

	Case 1: Healthy Schools in St. Louis, MO	Case 2: Behavioral Health Improvement in Washington, DC	Case 3: Youth Summit for Emotional Support in St. Louis, MO
Community and problem context	Regional efforts to promote healthy and successful schools, focusing on the challenge of integrating school districts, community organizations, and community voices around multiple definitions of health	Youth violence and suicide led to city‐wide commitment to improving student behavioral health. Lack of agreement across stakeholders on strategy and tactics	Regional call for systems change to address racial disparities in education and health outcomes. High school fellows from 9 districts chose to focus on addressing increases in teen suicide and depression in their schools
Core modeling team composition	Two core modeling teams working in parallel consisting of: one staff member of the Social System Design Lab; one faculty member from the Brown School; one staff member of Health Equity Works; 2 staff representatives from each school district, one representative of a community organizing working in the school district	One staff member of the Social System Design Lab; 2 faculty members and one staff member from George Washington University; one representative of a funding agency; one representative of the regional coordinating council	One staff members from the Social System Design Lab; One teacher from an area school district; 2 college‐aged program assistant who were former participants in the Changing Systems program
CBSD activities	Sets of 2 stakeholder workshops in 2 local school districts with planning and model synthesis by separate CMTs for each district	Two stakeholder workshops with planning and model synthesis by CMT	A 4‐day convening of high school students with planning and model synthesis by cohort of high school “fellows”
How did the workshop help integrate local context and WSCC framework?	Structured group model building activities provided a platform for participants to identify variables relevant to the local district priorities, then trace the causal relationships across diverse domains. Specific district‐related structures included interconnection between student mental health, disciplinary practices, and teacher wellbeing; recognition of the interconnection of student hunger, attendance, and performance	Using CLD as a boundary object to make discussion concrete, participants linked institutional racism and racial bias to discipline and access to behavioral health services. The group determined that historical racism and current racial tension during rapid gentrification need to be recognized when designing interventions	Students representing diverse schools in the region reflected on how inequities in district funding and resources by generate disparities in staff skills and wellness, influencing student skills and wellness, disparities in quality of school environment, and academic outcomes
How did participants integrate systems thinking?	Initial causal mapping activities and model synthesis review highlighted feedback relationships around parental engagement and tradeoffs of multiple strategies to support financial wellbeing at home. Additionally, discussion and models highlighted how accumulated history in school contexts are drivers of path dependence in parental engagement and trust	During discussion of CLD, participants recognized trust as a stock depleted by negative interactions, impairing future family and student engagement with school behavioral health promotion Understanding of competing resource demands across MTSS Emerging discussion of impact of starting position on system function	Students articulated the reinforcing relationship among academic stress, mental health, and physical health; Students explored structural barriers to strong student‐teacher relationships, peer relationships, and quality school environments as distinct from individual instances of malice or lack of concern
How did participants develop common language?	Through the negotiation of shared “boundary objects” during modeling sessions, diverse stakeholder groups convened on leadership flexibility, parental engagement, and parental trust as key leverage points for promoting child learning and wellbeing	Lengthy debate about the function of discipline in the CLD during workshop led to agreement on meaning of “exclusionary discipline” and role in damaging behavioral health	Extensive negotiation of the definitions of “good” and “bad” stress illuminated a deeper structural understanding of stress as an accumulation that influences academic performance and relationships in different ways
How did the CBSD process support mobilization for action?	The group model building activities facilitated the generation of action ideas negotiated by stakeholder groups, including “low‐hanging fruit” interventions; Since these workshops, partners at Health Equity Works have convened ongoing practice group of school districts to explore CBSD approaches	After reflecting on the social construction of systems thinking through the CBSD workshops and how “who is in the room” drives understanding, stakeholders called for expansion of participants to include students, parents, and teachers	At the close of the Changing Systems summit, youth led interactive presentations and discussions of calls to action with community stakeholders and disseminated policy briefs outlining these calls to action

CBSD, Community‐Based System Dynamics; CMT, Core Modeling Team; WSCC, Whole School, Whole Community, Whole Child; CLD, Causal Loop Diagram; MTSS, Multi‐Tiered System of Supports.

## RESULTS

### Case 1: Together for Healthy and Successful Schools in St. Louis, MO

A pressing concern for educational attainment within the St. Louis, MO, region includes the multiple and overlapping concerns of physical health, emotional wellbeing, poverty, and school quality. In 2018, Health Equity Works at the Brown School at Washington University in St. Louis undertook a research study in collaboration with the Brown School's Social System Design Lab (SSDL), with support of the Robert Wood Johnson Foundation, to explore innovative approaches for supporting implementation of the WSCC framework in local school districts, engaging school districts in the St. Louis region as research partners. CBSD represented one of a set of approaches designed to make explicit the interconnections, dependencies, and feedback relationships within the WSCC framework through a stakeholder‐driven, participatory process. The objectives of the workshops were to: (1) elucidate the underlying social system factors that determine healthy school environments in the St. Louis region, (2) develop a causal map focused on the interconnections between these factors that promote healthy and successful schools, and (3) identify changes, policies, and innovations that can be implemented in schools based on this map. A more comprehensive discussion of this overall Together for Healthy and Successful Schools project is available elsewhere in this issue.^30^

The Core Modeling Team for the Together for Healthy and Successful Schools project included school‐building staff and district leaders from 2 local school districts, the faculty principle investigator and a project coordinator from the research team, and a staff modeler and student research assistant from the SSDL. The design process started with a 3‐hour scoping and demonstration workshop conducted at a district elementary school that engaged core modeling team members from both districts as participants. Following an extensive planning and design process with the core modeling team, including a complete 3‐hour demonstration workshop to orient core modeling team members to the concepts and principles of CBSD, a series of community workshops were conducted in the 2 school districts. The first workshops focused on engaging participants (administrators, students, teachers, parents, and community partners) in developing a preliminary hypothesis of the system structure in small groups. In the second workshops, participants returned to critique and revise a synthesized model and to brainstorm and prioritize intervention points based on the model.

**Figure 2:**
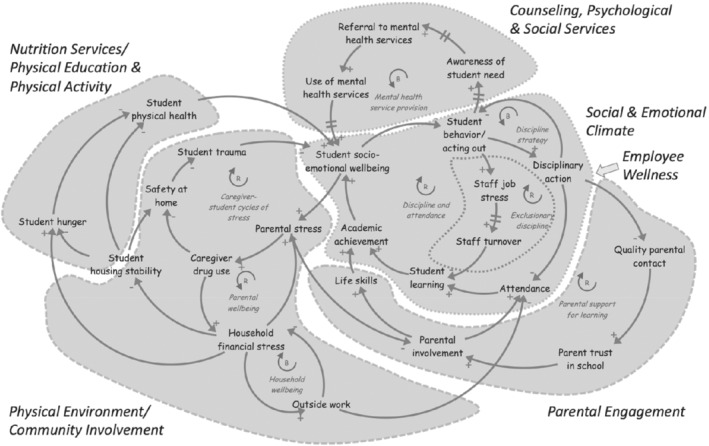
Causal Loop Diagram Artifact from Together for Healthy and Successful Schools Workshops, Overlaid with Components of the WSCC Framework

The modeling activities in the community workshops provoked dialog across stakeholder roles and resulted in a visual representation of the intersection of WSCC components, tailored to each district. Figure 2 provides a synthesis CLD from one district workshop sequence, highlighting the overlay of WSCC components.

In the district that produced the diagram in Figure 2, a series of conversations around persistently high rates of emotional distress among students engaged with school outreach specialists, school administrators, grandparents, and representatives from community organizations. Participants described how the predominance of disciplinary issues in parents‐school interactions undermined the trust needed to partner to promote student health. This distrust was layered on top of a deeper history of distrust of schools by many parents based on their own experiences as students in the same school districts a decade before. The insight stemming from this dialog and contribution to model structure was that current approaches to parental outreach ignored the intergenerational transmission of trust and memory within a school district, or the competing demands for attention and time of families and caregivers. Although the specific insight was not revolutionary to any one stakeholder group, the articulation of that challenge, and the prioritizing of updating how parental outreach was conceptualized, took a different and new form than had previously been addressed by any 1 group.

In this project, the CBSD workshops were embedded within a larger policy engagement process, in which the insights, common language, and social connections generated by the workshops were inputs to a series of message testing, scoping reviews, and social network analysis to identify district‐specific levers for change. CBSD contributed to: (1) revealing differences in mental models among stakeholder groups, including administrators, teachers, school staff, caregivers, and students about behaviors and policies that drive health in schools; (2) developing common language around mechanisms for promoting through which healthy and successful schools; and (3) building connections across schools and stakeholders to implement change, which continues past the conclusion of these workshops through the convening of a learning community exploring systems thinking approaches in their own schools.

### Case 2: A School Behavioral Health Stakeholder Learning Community in Washington, DC

Despite the fact that more than 95% of children in Washington, DC, have health care coverage, more than half do not receive the behavioral health treatment they need. One out of 6 (16%) high school students in DC reported attempting suicide in the last 12 months, which is more than double the national prevalence (7.4%).^31^ Expanding school behavioral health services has been high on the political agenda since the DC City Council unanimously passed the South Capitol Street Memorial Amendment Act in 2012, which mandated access to behavioral health services for all students in DC public schools and public charter schools in response to a shooting incident that killed 4 adolescents in 2010.

Efforts to fulfill the mandates of the 2012 Act and subsequent 2016 Youth Suicide Prevention Act have demonstrated both a broadly shared commitment to achieving universal access to high‐quality behavioral health services and an equally broad range of views on the best strategy to move forward. The Bainum Family Foundation had been investing for a number of years to increase access to high quality education in areas of the city in which children face higher levels of adversity. They and partners at the Center for Health and Health Care in Schools (CHHCS) at the George Washington University recognized the need to strengthen cross‐sector collaboration and to better partner with family and youth in DC to create a common vision for a city‐wide comprehensive school behavioral health system. Bainum and CHHCS formed the School Behavioral Health Stakeholder Learning Community (SLC) with school administrators, behavioral health providers and a provider association, children's health and legal advocates, parent advocates, and children's health researchers.

Initial meetings of the SLC emphasized the need for a structured approach to develop a shared vision as well as a demand for systems thinking. Bainum and CHHCS partnered with the SSDL to use CBSD to achieve the following goals: (1) develop a shared language to define a comprehensive school behavioral health system, (2) synthesize stakeholder perspectives on barriers to child behavioral health, and (3) build a foundation for the SLC to create a mission and identify leverage points for intervention. During the planning phase with the core modeling team, the SSDL provided training to CHHCS staff with the goal of leading future CBSD workshops. A CLD emerged from the 2, 3‐hour group model building workshops with the SLC that led to insights about the ways and the degree to which behavioral health needs are embedded in the broader context of the school and the community in Washington, DC.

The use of exclusionary discipline, in part to manage the problems caused by delays in appropriate behavioral health services, emerged during the workshops as one of the core barriers to meeting children's behavioral health and academic needs. The term “exclusionary discipline” as distinct from immediate efforts to resolve urgent safety issues emerged as common language from a lengthy and charged discussion about how to structure the CLD to capture the tension between maintaining a safe environment for all students and meeting individual students' behavioral health and educational needs. Exclusionary discipline used in the place of behavioral health services erodes the trust between students, families, and schools, which then serves as a further barrier to achieving educational goals and promoting wellbeing. This loss of trust and the resulting damage to student outcomes can be compounded by racial bias in the application of exclusionary discipline. By revising the CLD in real time during the discussion of this charged topic, workshop participants remained focused on how the structure of the system connected the experience of racial bias, exclusionary discipline, loss of trust, and lack of access to behavioral health services in a series of feedback loops with limited balancing loops within the system of a given school. One of the participants noted that: “When it goes well, it goes really well, and when it goes bad, it goes really bad.”

At the conclusion of the second workshop, the SLC participants expressed a desire to engage more diverse voices (parents, teachers, students, school leaders, and government agency leadership). In an ongoing second phase of the project, group model building workshops were held separately with high school students, parents, and teachers. The SLC plans to use a synthesized model to solicit input from stakeholders in the local philanthropic community and city government.

By critiquing and revising the CLD, members of the SLC gained deeper insight into the divergence of the group members' mental models of the complex system leading to persistent unmet behavioral need. Focusing on revising the CLD allowed the group to engage in extensive, yet productive discussion using a shared language. The renewed focus coming out of the SLC workshops on engaging new communities with direct involvement in school behavioral health in productive debate and development of shared understanding is one of the most important effects of this use of CBSD to mobilize community action for school behavioral health.

### Case 3: The Changing Systems Youth Summit on Emotional Support in Schools in St. Louis, MO

In the racially segregated St. Louis, MO, metropolitan area, the largest predictors of child's education and health outcomes are their zip code and the color of their skin.^32^, ^33^ The Ferguson Commission Report, written in response to the 2014 murder of Michael Brown, Jr. by a police officer, calls for urgent and regional systems change to close racial disparities.^34^ The report's signature priority of “Youth at the Center” calls for educational reform that meaningfully engages the stakeholders most impacted and avoids the fragmentation that characterizes the region. While high school students are embedded in education systems every day, there are few opportunities for students from different districts to come together and solve problem on equity issues in education.

During 2015–2018, high school fellows with the SSDL at the Brown School of Social Work have used CBSD to design and facilitate the Changing Systems Youth Summit, a 4‐day, youth‐led convening on issues such as structural racism in schools, youth homelessness, and gun violence. Changing Systems seeks to understand how St. Louis youth from across economic and racial divides in St. Louis understand, process, and experience social justice issues, while building their capacity in systems thinking and system dynamics. Ultimately, the mission of Changing Systems is to equip St. Louis youth to advance social justice in their schools and communities through systems thinking, leadership, and advocacy skills.

In June 2019, 24 youth from 9 school districts in the St. Louis region convened for a 4‐day, youth‐led summit on a complex community issue of their choosing, “Emotional Support in Schools.” Of these youth, 13 high school fellows facilitated the summit after 3 weeks of training, co‐design, and application of system dynamics tools.

During the summit, SSDL Fellows engaged their peers in by facilitating CBSD workshops to identify factors that affect the students' experience with emotional support in schools, to understand the underlying system structure, to brainstorm intervention points based on the model, and to create advocacy plans. On the final day, fellows and participants presented model‐informed calls to action to an audience of 35 community stakeholders, leading an interactive “World Café” dialog to engage youth and adults in discussing results and implications for area schools. Following the summit, fellows continued to work with the causal maps produced by summit participants to finalize a model that describes school mental health from the perspective of regional high school students. Ultimately, Fellows produced and disseminated a policy brief with 4 calls to action for meeting the mental health needs of Missouri students: (1) integrating emotional literacy into curriculum, (2) changing the grading system to adapt to individual learning paces, (3) implementing “Peace Rooms” in every school as a private space for students to be alone and destress, and (4) increasing awareness of emotional support resources in school.

In Changing Systems, CBSD is an avenue for young people to engage in critical dialog, make meaning of the complex problems they experience, and collaborate with other actors in the system to design solutions. Youth serving as peer leaders and program co‐designers create an environment for students to speak out openly and honestly about their experiences, to learn from a wide range of student experiences and mental models across the region, and to empower individual students to create change in their own schools and communities. In post‐surveys completed by participants, 94% of participants agreed that system dynamics is useful to understand problems and develop solutions; 95% agreed that they saw the issue of emotional support from a different perspective than one they previously had; 85% of participants felt this experience improved their ability to make a difference in their school or community; and 100% of participants felt this experience provided a valuable experience for youth to have their voices heard. The Changing Systems Summit on Emotional Support as Schools suggests that CBSD has potential to meaningfully engage and mobilize young people with diverse educational experiences as partners in regional systems change.

### Cross‐Case Synthesis

Although the 3 cases come from 2 different cities and have varying degrees of focus on youth behavioral health, they share the participants' explicit recognition of the role that racism, poverty, and violence play in shaping the system of their school environments. In all 3 cases, participants were able to address how this broader context influenced the school system to identify potential changes within schools needed to respond to these forces.

One underlying assumption across the 3 cases is that increasing participants' understanding of how complex systems function would improve their ability to engage in productive dialog with other participants about the problems and potential solutions embedded in the CLD. In Case 1 and Case 2, participants identified the time lag between experiences with schools and trust in the school institution by students and families. In Case 1, the persistent deficit in trust spanned generations. Declines in trust from negative school experiences also served as important variables in feedback loops in these models. In both cases, including trust as an explicit variable and working through how the time lags and feedback loops functioned led to new participant insights into the challenges of family engagement in the context of the experience of racism and disinvestment in schools and other resources. In Case 3, youth participants identified feedback loops between their classroom experiences and their own ability to seek support and form relationships with teachers and peers, leading to new insights into the specific training and spaces they need to understand, process, and communicate their emotions at school. In all 3 cases, pressures on teachers impeded their ability to form relationships and build trust with students and families, further exacerbating teacher stress.

The development of common language to describe distinct experiences of the system dynamics across participants was a result of working together to understand and revise the CLD representation of the system dynamics. This is consistent with the underlying theory of CLDs as *boundary objects* in collaborative problem‐solving efforts.^29^ In Case 2, the participants moved from a discussion of “discipline” to “exclusionary discipline” in order to resolve debate about the dependencies linking discipline and student behavioral health. This language shift was a critical output of the workshop due to its function within the group's constructed meaning of the system as well as its link to a broader policy debate in Washington, DC, and nationally regarding exclusionary discipline.

The patterns of mobilization to action varied across cases more than the themes related to the other research questions. In Case 1, the outputs of the CBSD process were used as inputs to an ongoing policy development process. In Case 2, the stakeholders had a strong preference for moving quickly to policy recommendations but determined during the process that engaging with a broader set of stakeholders in the city was required to represent aspects of the system that were not elicited in the 2 workshops. In Case 3, the Youth Summit resulted in immediate outreach to other stakeholders during an in‐person meeting as well as development of a policy brief disseminated to local policymakers.

## DISCUSSION

Community‐based system dynamics provides a structured approach to engaging community voice in the design and facilitation of participatory modeling exercises that make explicit the complex interconnections embedded in the WSCC framework. Here we discuss potential contributions and limitations of CBSD approaches to operationalizing concepts of school health within districts and school buildings.

### Centering Voices of Those Most Impacted by Policy Interventions

One of the central challenges of implementing a WSCC framework is centering the voices and perspectives of people embedded in the system.^5^, ^35^ An underlying principle of system dynamics approaches is that quantitative data provide only 1 small source of knowledge of how real people make decisions, what information cues are used, and the sources of delays. The mental database of those living, working, and making daily decisions in a system is a vastly richer source of information about system structure.^36^ In the cases presented above, the structured CBSD approach prioritized engaging the voices of stakeholders who are conventionally left out of problem formulation and policy‐making processes. By creating activities that elicit lived experiences about complex and interconnected aspects of school health from these stakeholders, the themes that emerge suggest prioritizing action outside of conventional domains of school health intervention. These nuanced potential leverage points, such as explicit outreach to parents and caregivers to repair damaged relationships and distrust of school, restructuring disciplinary practices to explicitly focus on racial biases in applications of exclusionary discipline, and focusing on student self‐regulation and emotional awareness rather than exclusively on service provision, are specific to the districts highlighted in these projects, but may suggest priorities for other districts seeking to center those who are most impacted by policy decisions.

### Building Capabilities Internally and Partnering to Acquire Expertise

Each of the cases in this study emerged from different funding scenarios, community contexts, and problem domains, with different objectives and conceptions of impact. Yet, there are consistent principles and conditions for engaging in CBSD approaches support implementation of the WSCC framework. First, the process depends on a core group of community leaders and formal institutional leaders who are committed to a process of inquiry, community engagement, and learning that by definition does not have a preordained conclusion. All 3 cases invested in explicit activities around language negotiation and learning around concepts of systems thinking and system dynamics. Second, a collaboration between individuals with local contextual and subject matter expertise must be in dialog with facilitators or modelers with methods expertise in system dynamics modeling. In all 3 cases, a core modeling team of district stakeholders and modelers was convened to co‐design, co‐facilitate, and co‐develop insights and recommendations. District stakeholders took on modeling roles in sessions, and modelers invested in learning the language and priorities of the local context. Third, a trusted community stakeholder is necessary to extend their social and political connections to convene stakeholders, helping to create an open space where stakeholders can openly and critically reflect on their experiences of system structure. In all 3 cases, specific efforts were taken to identify the individual or organization that could serve as a trusted convener—in all cases these conveners had deep roots in school communities and had an expectation of long‐term collaboration after the modeling efforts concluded.

### Limitations and Methodological Challenges to Assessing Impact of CBSD Approaches

Just as the WSCC framework is not intended to be a panacea that leads to change on its own, implementation of CBSD in support of WSCC does not guarantee successful or impactful implementation. In all 3 cases, CBSD approaches were embedded within larger change efforts. Qualitative description can convey the expanded boundaries of participation in decision‐making to include voices that are typically left out of policy‐making processes, the capacity building within stakeholder groups to analyze underlying sources of resistance and barriers to change, and the development of new language and understanding of intervention priorities. Yet, it is often difficult to isolate the specific contribution of CBSD to policy changes to make firm assessments of attribution; as in other community‐based work, it is difficult to know for certain what would have happened if a different approach had been taken. Whereas there is a growing literature of case studies of varying methodological rigor, evaluation studies, and tools for informing the design of CBSD work,^37^, ^38^, ^39^, ^40^ there is no standardized, evidence‐based protocol for implementing CBSD approaches in schools or otherwise. These limitations and conceptual challenges are not intended to dissuade school districts, schools, or community organizations from undertaking a CBSD approach to supporting implementation of the WSCC framework, but rather to understand that this is a process‐intensive, reflective, and long‐term undertaking.

### IMPLICATIONS FOR SCHOOL HEALTH

Realizing frameworks such as the WSCC model in schools presents a complex systems problem that requires tools for engaging with complex systems. The results of these 3 case studies suggest that CBSD is a promising approach for building shared understanding and ownership among stakeholders in a system, tailoring frameworks to unique community contexts and dynamics, and mobilizing stakeholders for action by building capacity to understand, and therefore change, systems. Based on these reflections we recommend:

- Invest dedicated time and human resourcing in developing capabilities for systems thinking and system dynamics perspectives within districts. These investments can and should be made in collaboration with community organization and/or university partners, but they are the foundations on which the community engaged approaches described here are built.
- Prioritize engagement by those who are conventionally left out of problem scoping and formulation processes; students, caregivers, school support staff, and administrative team members have unique insight into the underlying structural barriers to implementing school health policy interventions.
- Structured and intentionally designed processes can support, but not guarantee, the management of power dynamics across stakeholder groups (such as superintendents, teachers, parents and caregivers, students, administrators, and representatives from community organizations). The deep, reflective work of considering the underlying structure supporting school health requires working in teams to integrate facilitation and community engagement skills, familiarity with the science and best practices in policy, and capabilities in modeling and systems analysis.
- Approach the work of developing contextually driven, locally relevant vision of the WSCC framework as a practice. There are resources, best practices, and tools available to support this work, but also opportunity for innovation and experimentation to integrate new methods, tools, and approaches into this work.

### Human Subjects Approval Statement

The projects reported on in this paper are not considered to be human subjects research and therefore did not require human subjects research approval.

### Conflict of Interest

Dr. Long is a member of the American School Health Association Research and Publications Committee and the Editorial Advisory Council of the Journal of School Health. He had no role in the selection or review of this paper and has no other conflicts of interest. All other authors of this article also declare they have no conflicts of interest.
